# Intracellular Fate of a Dual-Fluorescent Hydrophobic Ion Pair: Comparison of Lipid-Based Nanocarriers

**DOI:** 10.1021/acs.molpharmaceut.5c01633

**Published:** 2026-02-12

**Authors:** Gabriela Koutná, Lena Werner, Martyna Truszkowska, Luca Maurice Richter, Kateřina Kubová, Andreas Bernkop-Schnürch

**Affiliations:** 1 Department of Pharmaceutical Technology, Institute of Pharmacy, 27255 University of Innsbruck, Innrain 80/82, Innsbruck 6020, Austria; 2 Department of Pharmaceutical Technology, Faculty of Pharmacy, 37748 Masaryk University, Palackého třída 1946/1, Brno 612 00, Czech Republic

**Keywords:** fluorescent hydrophobic ion pair, self-emulsifying drug delivery system, nanoemulsion, liposomes, intracellular fate, confocal laser scanning microscopy

## Abstract

Effective intracellular trafficking and delivery of hydrophilic drugs remain challenging due to poor membrane permeability and limited encapsulation in lipid-based nanocarriers. To address this, we developed a dual-fluorescent hydrophobic ion pair (HIP) by pairing a model fluorescent hydrophilic drug, Cascade Blue hydrazide, with the lipophilic probe DiA. The HIP was subsequently incorporated into three lipid-based nanocarriers—self-emulsifying drug delivery systems (SEDDS), nanoemulsions, and liposomes—to enable visualization and comparison of how formulation composition influences intracellular uptake and fate of a model hydrophilic drug surrogate delivered as an HIP complex. The complex showed a precipitation efficiency of 95% and an >8130-fold increase in lipophilicity compared to noncomplexed Cascade Blue hydrazide, which enabled incorporation into SEDDS (64.41 ± 0.26 nm), nanoemulsions (92.61 ± 1.27 nm), and liposomes (175.03 ± 3.18 nm). Dissociation studies revealed a strong medium dependence, with <10% release in FaSSGF but ∼60% in phosphate-rich FeSSIF. Cytotoxicity testing demonstrated >90% cell viability at 0.01% for all formulations after 24 h, confirming their biocompatibility under relevant conditions. Hemolysis assays showed negligible membrane disruption for SEDDS, while uptake studies in Caco-2 cells indicated that internalization was mainly energy-dependent, with modest effects observed after inhibition of clathrin- and caveolae-mediated pathways. Confocal laser scanning microscopy highlighted a formulation-dependent intracellular fate: SEDDS confined Cascade Blue to vesicular compartments while redistributing DiA to the plasma membrane, whereas nanoemulsions and liposomes enabled endosomal escape, dispersing Cascade Blue into the cytosol and relocating DiA to perinuclear and plasma membranes. Liposomes also showed residual uptake at 4 °C with membrane colocalization of DiA, supporting fusion as a complementary uptake mechanism.

## Graphical abstract

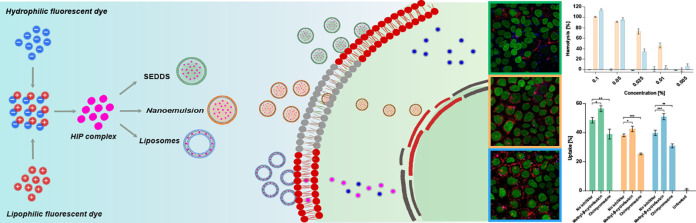

## 1 Introduction

The intracellular delivery of drug-loaded nanocarriers is a complex, multistep process governed by various cellular uptake mechanisms and strongly influenced by the physicochemical properties of the nanoparticles.^1–3^ To achieve successful therapeutic outcomes, the nanocarriers must cross biological barriers, permeate the cell membrane, and ultimately reach the intracellular site where the target resides.^1,4^ Cellular entry occurs via both energy-dependent and energy-independent processes. Among the energy-dependent routes, endocytosis represents the predominant pathway and includes clathrin-mediated, caveolae-mediated, macropinocytic, and phagocytic processes.^5,6^ In contrast, membrane fusion is an energy-independent mechanism that allows the cargo to be delivered directly to the cytosol, thereby minimizing lysosomal degradation in the acidic, enzyme-rich environment of endosomes and lysosomes.^4,7,8^

Fluorescent dyes and fluorescently labeled molecules are commonly used to track the intracellular fate of nanocarriers.^9–11^ However, studying hydrophilic drugs remains particularly challenging due to their high aqueous solubility, poor membrane permeability, and limited encapsulation efficiency in lipid-based systems.^12^ Hydrophobic ion pairing (HIP) has emerged as a simple yet effective strategy to overcome these limitations. By pairing a hydrophilic drug with an oppositely charged counterion, HIP reduces aqueous solubility and improves drug incorporation into lipid-based carriers. Once inside the cell, the ion pair dissociates in the presence of competing ions, restoring the native hydrophilic drug.^13^ This dual behavior—improving formulation compatibility while ensuring intracellular release—makes HIP an attractive tool for mechanistic studies of drug delivery. In this work, a hydrophilic fluorescent probe Cascade Blue hydrazide was used as a model hydrophilic drug. To enable its incorporation into lipid-based nanocarriers and to allow simultaneous fluorescent tracking, it was paired with the lipophilic probe DiA to form a dual-fluorescent HIP complex, thereby increasing the apparent lipophilicity of the hydrophilic model drug and enabling visualization of the HIP within the formulation, during uptake and following intracellular release.

In previous work done by our group, a dual-fluorescent HIP complex was incorporated into nanoemulsions and studied using confocal microscopy, where both dyes of one HIP system could be visualized at the cellular membrane.^14^ Building on these findings, we aimed to extend this concept beyond a single formulation type and to gain a deeper understanding of how carrier composition influences intracellular delivery pathways. The aim of this study was therefore to compare how different lipid-based nanocarriers—self-emulsifying drug delivery systems (SEDDS), nanoemulsions, and liposomes—with distinct compositions affect the cellular uptake, intracellular fate, and localization of the HIP complex. Liposomes were primarily composed of fusion-promoting lipids (DOPE, cholesterol, and DOTAP), which possess high intrinsic negative curvature and a positive surface charge known to facilitate membrane fusion.^7,15^ This composition was chosen to explore whether fusion processes could also occur during internalization. Nanoemulsions were composed of both fusogenic and nonfusogenic components, while SEDDS contained only nonfusogenic excipients. This design enabled us to systematically explore how formulation composition influences intracellular delivery pathways and the fate of HIP-loaded systems following internalization.

## 2 Materials and Methods

### 2.1 Materials

Cascade Blue hydrazide trisodium salt (8-methoxypyrene-1,3,6-trisulfonic acid, trisodium salt, hydrazide derivative), DOPE (1,2-dioleoyl-*sn*-glycero-3-phosphoethanolamine), Tween 80 (polyethylene glycol sorbitan monooleate), Cremophor EL (castor oil polyoxyethylene ether), isopropyl alcohol, chlorpromazine, methyl-β-cyclodextrin, trypan blue, and dialysis membrane Spectra-Por Float-A-Lyzer G2 (MWCO 20 kDa) were obtained from Sigma-Aldrich (Vienna, Austria). Cholesterol and oleic acid were purchased from Thermo Fisher GmbH (Kandel, Germany). DiA (4-(4-dihexadecylaminostyryl)-*N*-methylpyridinium iodide)) and NucSpot Live 650 were obtained from Biotium (California, USA). Labrafac Lipophile WL 1349 (medium-chain triglycerides) was received as a gift from Gattefossé (Lyon, France). DOTAP-Cl (1,2-dioleoyloxy-3-trimethylammonium-propane chloride) was obtained as a free sample from Lipoid GmbH (Ludwigshafen, Germany). Solutol HS-15 (polyethylene glycol-15-hydroxystearate) was purchased from BASF. Capmul MCM (medium-chain mono- and diglycerides) was obtained from Abitec Corporation (Columbus, USA). 3F powder for the preparation of simulated fluids was obtained from Biorelevant (London, UK). All other chemicals were purchased from Sigma-Aldrich (Vienna, Austria) or other commercial sources.

### 2.2 HIP Preparation

Hydrophobic ion pairs between two fluorescent dyes were prepared using the Bligh–Dyer method, as previously described.^14^ Cascade Blue hydrazide was dissolved in demineralized water at a concentration of 50 μg/mL. DiA was dissolved in chloroform at various concentrations to achieve charge ratios of 0.5:1, 1:1, and 2:1 (DiA:Cascade Blue). For each preparation, 250 μL of the Cascade Blue hydrazide solution was mixed with 250 μL of the DiA solution, followed by the addition of 500 μL of methanol to form a Bligh–Dyer monophase. The mixture was then incubated at 750 rpm and 25 °C for 45 min in the dark using a thermomixer (ThermoMixer C, Eppendorf Vertrieb Deutschland GmbH, Germany). Subsequently, 250 μL of demineralized water and 250 μL of chloroform were added to induce phase separation. The resulting HIP complex was extracted into the chloroform phase. The aqueous phase was carefully removed, and 100 μL was analyzed for a noncomplexed hydrophilic dye via absorbance at 402 nm using a Spark plate reader (Tecan Spark, Tecan Trading AG, Switzerland). The chloroform was then evaporated at 60 °C, and the resulting complexes were freeze-dried overnight (Christ Gamma 1-16 LSC Freeze-Dryer, Martin Christ Gefriertrocknungsanlagen GmbH, Germany).

The precipitation efficiency (PE) of the complex was calculated using the following formula:

$$\mathrm{PE}\left[\%\right]=100-\frac{c\;\mathrm{of}\;\mathrm{Cascade}\;\mathrm{Blue}\;\mathrm{after}\;\mathrm{HIP}}{c\;\mathrm{of}\;\mathrm{Cascade}\;\mathrm{Blue}\;\mathrm{before}\;\mathrm{HIP}}\times 100$$

### 2.3 Evaluation of HIP

The partition coefficient of Cascade Blue hydrazide and HIP between *n*-octanol and water was determined to assess the lipophilicity of the HIP complex. Cascade Blue hydrazide (12.5 μg) and HIP (prepared as described in Section 2.2) were dissolved in 500 μL of a 1:1 *n*-octanol:water mixture. The samples were then incubated at 37 °C and 300 rpm, protected from light, for 24 h using a thermomixer. After incubation, the phases were separated by centrifugation (MiniSpin, Eppendorf AG, Germany) at 12,500 rpm for 10 min.^16^ Subsequently, 100 μL of the aqueous phase was collected, and the concentration of Cascade Blue was determined by absorbance at 402 nm using a Tecan Spark plate reader. The corresponding amount in the organic phase was calculated accordingly. The log *P* value was then calculated using the following equation:

$$\log P_{n‐\mathrm{octanol}/\mathrm{water}}=\log\;\frac{c\;\mathrm{of}\;\mathrm{Cascade}\;\mathrm{Blue}\;\mathrm{in}\;n‐\mathrm{octanol}}{c\;\mathrm{of}\;\mathrm{Cascade}\;\mathrm{Blue}\;\mathrm{in}\;\mathrm{water}}$$

The excitation and emission fluorescence spectra of Cascade Blue, DiA, and the resulting HIP complex were recorded by dissolving each component in demineralized water containing 1% DMSO. Additionally, fluorescence measurements were conducted to investigate potential Förster resonance energy transfer (FRET) interactions between the fluorescent dyes. This combined analysis provides detailed insights into the spectral properties and intermolecular interactions within the multidye system.

### 2.4 Dissociation of HIP

The dissociation of the fluorescent HIP was evaluated in several different media, including demineralized water, 5% NaCl, 5% KCl, 5% CaCl_2_, fasted-state simulated gastric fluid (FaSSGF), fasted-state simulated intestinal fluid (FaSSIF), and fed-state simulated intestinal fluid (FeSSIF). Freeze-dried HIP (prepared as described in Section 2.2) was reconstituted by adding 10 mL of each test medium. The samples were then incubated at 37 °C and 400 rpm in a thermomixer protected from light for 24 h. At predetermined time points, 100 μL aliquots were withdrawn from each sample. The fluorescence intensity of Cascade Blue in these aliquots was measured at an excitation wavelength of 330 nm. All experiments were performed in triplicate.

### 2.5 SEDDS, Nanoemulsion, and Liposome Preparation

SEDDS and nanoemulsion formulations were prepared by homogenizing the excipients listed in Table 1 at 75 °C and 1000 rpm for 1 h using a thermomixer to produce the preconcentrates of SEDDS and nanoemulsions.

**Table 1:** Composition of SEDDS, Nanoemulsion, and Liposomal Formulations, Expressed as Weight Percent (w/w; %)

**formulation**	**components**	**[%]**
SEDDS	medium-chain triglycerides	50
	polyethylene glycol-15-hydroxystearate	30
	glyceryl monocaprylate	20
nanoemulsion	1,2-dioleoyl-*sn*-glycero-3-phosphoethanolamine	8
	cholesterol	3
	oleic acid	29
	castor oil polyoxyethylene ether	20
	polyethylene glycol sorbitan monooleate	20
	isopropylalcohol	20
liposomes	cholesterol	50
	1,2-dioleoyl-*sn*-glycero-3-phosphoethanolamine	10
	1,2-dioleoyloxy-3-trimethylammonium-propane chloride	6.7
	oleic acid	33.3

Liposomes were prepared using the ethanol injection method.^17^ The lipids (15 mg of cholesterol, 10 mg of oleic acid, 3 mg of DOPE, and 2 mg of DOTAP) were dissolved in 200 μL of ethanol at 70 °C and 1000 rpm using a thermomixer. The aqueous phase, consisting of 1% Tween 80, which served as a stabilizing agent,^18^ was heated up to 70 °C. The ethanolic lipid solution was quickly injected into the prewarmed surfactant solution and vortexed for 2 min. The mixture was then shaken at 500 rpm at 40 °C for 2 h to evaporate the ethanol. The formed liposomes were cooled down to 4 °C in a thermomixer and stored in the fridge until further use.

HIP-loaded SEDDS and nanoemulsions were prepared by dissolving fluorescent HIP in 50 μL of SEDDS/nanoemulsion preconcentrate with a final concentration of 237.5 μg/mL SEDDS/nanoemulsion preconcentrate. For the liposomes, the concentration of the HIP was 11.9 μg/mL of the lipid dispersion.

### 2.6 SEDDS, Nanoemulsion, and Liposome Characterization

All formulations were characterized for particle size and polydispersity index (PDI) via the dynamic light scattering (DLS) technique, and for zeta potential via electrophoretic light scattering, using a ZetaSizer Nano ZSP (Malvern Instruments, UK).

Blank and HIP-loaded samples were diluted to a final concentration of 1% (v/v) in HEPES-buffered saline (HBS) solution prior to analysis. For the SEDDS samples, gentle agitation was applied following HBS dilution to generate a fine nanoemulsion. The nanoemulsion samples were processed by vortexing for 30 s, followed by ultrasonication using a Hielscher UP200H (Hielscher, Germany) at a power of 10 W and an amplitude of 60% for 30 s, in order to reduce the particle size.

All samples were then incubated at 37 °C and 400 rpm in a thermomixer. Particle size and PDI were evaluated at 0, 2, 4, 6, and 24 h postincubation. The stability of each formulation was also evaluated in biorelevant fluids, including FaSSGF and FaSSIF. All samples were analyzed in triplicate. The stability of the formulations was also evaluated by incubating the samples at 4 °C to ensure their stability for further experiments conducted at low temperatures.

The distribution coefficient (log *D*) between the SEDDS/nanoemulsion and the release media (HBS at pH 7.4; FaSSGF at pH 1.6) was also evaluated. HIPs (59.4 μg) were incubated with 50 μL of SEDDS/nanoemulsion preconcentrate and with HBS/FaSSGF. The samples were then incubated in a thermomixer at 600 rpm and 37 °C for 24 h. Following incubation, the samples were centrifuged at 12,500 rpm for 10 min. Subsequently, 10 μL of the supernatant was withdrawn, diluted with methanol to a final volume of 1 mL, and analyzed. The amount of dissolved Cascade Blue was then analyzed using a Tecan Spark plate reader to measure fluorescent intensity at an excitation wavelength of 330 nm.

$$\log\;D=\log\;\frac{c\;\mathrm{of}\;\mathrm{Cascade}\;\mathrm{Blue}\;\mathrm{in}\;\mathrm{SEDDS}/\mathrm{nanoemulsion}}{c\;\mathrm{of}\;\mathrm{Cascade}\;\mathrm{Blue}\;\mathrm{in}\;\mathrm{release}\;\mathrm{medium}}$$

### 2.7 Drug Release Studies

The release of Cascade Blue hydrazide from the formulations was evaluated using a modified membrane diffusion method.^19^ The HIP-loaded SEDDS, nanoemulsions, and liposomes were diluted with HBS buffer solution to a final concentration of 1% (v/v). As a control, a Cascade Blue hydrazide solution was prepared at a concentration of 2.5 μg/mL, matching that of the dye in the formulations. Aliquots of 1 mL were transferred into dialysis membranes (Spectra-Por Float-A-Lyzer G2) and dialyzed against 10 mL of HBS buffer at 37 °C with agitation (100 rpm) for up to 48 h. At predetermined time points, 100 μL samples were withdrawn from the release medium and immediately replaced with an equal volume of fresh HBS buffer. The amount of released Cascade Blue was quantified by measuring fluorescence intensity using a multiplate reader at 330 nm excitation and 425 nm emission wavelengths.

### 2.8 Cell Viability: Resazurin Assay

To evaluate the cytotoxic effects of the blank and HIP-loaded formulations, as well as of the inhibitors, a resazurin assay was performed according to a previously described method.^20^ Prior to the experiment, Caco-2 cells were seeded at a density of 5 × 10^4^ cells per well in 96-well plates and incubated for 3 days at 37 °C in a humidified atmosphere (95% humidity, 5% CO_2_), to allow the cells to reach confluency. A culture medium of Minimum Essential medium (MEM) supplemented with 10% (v/v) heat-inactivated fetal bovine serum (FBS) and penicillin/streptomycin (100 units/0.1 mg/L) was used.

At the start of the experiment, the culture medium was removed, and the cells were washed twice with sterile HBS. Then, 100 μL of the test samples was added to each well, and the plates were incubated at 37 °C for either 4 or 24 h. For these experiments, the concentrations of the SEDDS, nanoemulsions, and liposomes ranged from 0.5% to 0.01% (v/v) in sterile HBS at pH 7.4. To evaluate the cytotoxic effect of the inhibitors, concentrations ranging from 50 to 5 μM for chlorpromazine and from 50 to 5 mM for methyl-β-cyclodextrin were applied, followed by a 2 h incubation at 37 °C. Sterile HBS and 0.1% (v/v) Triton X-100 were used as the negative and positive controls, respectively.

After the incubation period, the cells were washed twice with sterile HBS, and 150 μL of 0.1% (w/v) resazurin solution was added to each well. Following a further 2 h incubation at 37 °C, 100 μL of the supernatant was transferred to a black 96-well plate and fluorescence intensity (FI) was measured using a Tecan Spark plate reader at an excitation wavelength of 540 nm and an emission wavelength of 590 nm.

Cell viability was calculated using the following equation:

$$\mathrm{cell}\;\mathrm{viability}\left(\%\right)=\frac{\mathrm{FI}\;\mathrm{of}\;\mathrm{the}\;\mathrm{sample}-\mathrm{FI}\;\mathrm{of}\;\mathrm{the}\;\mathrm{p. control}}{\mathrm{FI}\;\mathrm{of}\;\mathrm{the}\;\mathrm{p. control}-\mathrm{FI}\;\mathrm{of}\;\mathrm{the}\;\mathrm{n. control}}\times 100$$

### 2.9 From Uptake to Fate: Intracellular Behavior of HIP-Loaded Nanocarriers

#### 2.9.1 Cellular Uptake

Cellular uptake studies were conducted using the Caco-2 cell line and analyzed by flow cytometry, in accordance with previously published protocols.^14^ The Caco-2 cells were seeded at a density of 2.5 × 10^4^ cells per well in 24-well plates and cultured for 10 days at 37 °C in a humidified atmosphere (95% humidity, 5% CO_2_) until full confluency was reached. On the day of the experiment, the SEDDS, nanoemulsions, and liposomes were prepared as described above. The SEDDS, nanoemulsions, and liposomes were diluted with a sterile HBS buffer to a final concentration of 0.01% (v/v). Caco-2 cells were washed twice with sterile HBS buffer, followed by the addition of 500 μL of chlorpromazine (30 μM) and methyl-β-cyclodextrin (5 mM) solution in HBS buffer to selected wells as endocytosis inhibitors. Cells were incubated with the inhibitors at 37 °C for 45 min. Subsequently, cells were washed with sterile HBS buffer and incubated at either 37 and 4 °C for an additional 45 min. Following this incubation, 500 μL of the respective samples was added to each well, and cells were incubated for 2 h at 37 or 4 °C, protected from light. For experiments at 4 °C, all solutions were precooled prior to application. After incubation, the samples were removed, and cells were washed twice with sterile phosphate-buffered saline pH 7.4 (PBS). The cells were detached by adding 150 μL of trypsin/EDTA solution (0.05%/0.02%) and incubating at 37 °C for 5 min. The reaction was stopped by adding 400 μL of MEM and gently pipetting for 30 s to complete cell dissociation. The cell suspensions were transferred to 15 mL Falcon tubes and centrifuged at 800 rpm for 4 min. The supernatant was then discarded, and the cell pellet was resuspended in 6 mL of PBS and centrifuged again under the same conditions. Finally, the cells were resuspended in 400 μL of PBS, gently vortexed, and filtered through a 70 μm cell strainer. The strainer was then washed with an additional 400 μL of PBS. Samples were analyzed using an Attune NxT flow cytometer (Thermo Fisher Scientific, MA, USA). The fluorescence signal of 100,000 events was measured. Surface-attached fluorophores were quenched by addition of 50 μL of Trypan Blue (25 μg/mL) prior to fluorescence detection. The percentage of cells with high fluorescence DiA emission that took up formulations containing HIP was determined within the sorted population using a BL2-A channel (Ex = 488 nm, Em = 575 nm). Data were analyzed using FlowJo v10.8. Compensation for the two dyes was performed prior to flow cytometry to correct for fluorescence spillover effects.

#### 2.9.2 Endosomal Escape Studies

The ability of the formulations to escape from endosomes was assessed by measuring the release of hemoglobin from erythrocytes upon exposure to the tested formulations. The erythrocyte concentrate, kindly provided by Tirol Kliniken GmbH (Innsbruck, Austria), was suspended in a ratio of 1:200 (v/v) in sterile HBS buffer (pH 7.4).^21^ The concentrations of the SEDDS, nanoemulsion, and liposomal formulations ranged from 0.1% to 0.01% (v/v), while inhibitor concentrations ranged from 50 to 5 μM for chlorpromazine and from 50 to 5 mM in the case of methyl-β-cyclodextrin. HBS buffer and 0.5% (v/v) Triton X-100 served as negative and positive controls, respectively. Aliquots of the tested formulations (500 μL) were added to the diluted erythrocyte suspension at a 1:1 ratio and incubated at 37 °C and 150 rpm for 4 h (2 h for the inhibitors) in an orbital shaker incubator (ES-80, Grant Instruments Ltd., UK). After incubation, the samples were centrifuged for 10 min at 2700 rpm. Then, 100 μL of each supernatant was analyzed by measuring the absorbance at 415 nm using a Tecan Spark plate reader. The following equation was used to determine the percentage of hemolysis:

hemolysis(%)=[ABSofthesample−ABSofthen. control]/[ABSofthep. control−ABSofthen.
control]×100

#### 2.9.3 Intracellular Trafficking of the HIP Complex

To further evaluate the cellular uptake and intracellular distribution of the fluorescent HIP, confocal laser scanning microscopy (CLSM) (Leica TCS SP8, Leica Microsystems, Vienna, Austria) was employed. Caco-2 cells were seeded at a density of 1 × 10^5^ cells/mL (3 × 10^4^ cells/well) in 8-well Ibidi plates (Ibidi, Munich, Germany) and cultured for 3 days under the above-described conditions.^10^ For the experiment, all formulations were diluted in Opti-MEM to match the concentration used in the cellular uptake studies. Initially, the cells were washed twice with Opti-MEM. Then, 200 μL of chlorpromazine (30 μM) and methyl-β-cyclodextrin (5 mM) was added, after which the cells were incubated at 37 °C for 45 min. Subsequently, the cells were washed twice with Opti-MEM and stained with NucSpot Live 650 nuclear stain for 2 h at 37 °C. Following two further washes with Opti-MEM, cells were incubated at 37 or 4 °C for 45 min. The respective formulations (200 μL) were then added and incubated for a further 2 h. Following this incubation period, the formulations were removed and replaced with fresh Opti-MEM. For experiments conducted at 4 °C, all solutions were precooled prior to application. All fluorescence micrographs were acquired using identical imaging parameters. Image processing was conducted with ImageJ software. For *yz*- and *xz*-section visualizations, five consecutive *XY* planes from the *z*-stack (0.2 μm step size) were used to generate the projections. To prevent signal overlap between channels, crosstalk correction and spectral unmixing were applied to compensate for emission spectrum interference. In addition, Gaussian-based 2D filtering was used to refine the image quality.

### 2.10 Statistical Data Analysis

All experiments were performed at least in triplicates, and results were presented as means ± standard deviation (SD). Statistical analysis was performed via one-way ANOVA (GraphPad Prism 5) with *p* < 0.05 considered as the level of significance.

## 3 Results and Discussion

### 3.1 Hydrophobic Ion Pairing

Cascade Blue hydrazide, a hydrophilic compound bearing three negatively charged sulfonate groups, was complexed with the positively charged, lipophilic counterion DiA (Figure 1A), at three different charge ratios (0.5:1, 1:1, and 2:1) using the well-established Bligh–Dyer method. This method is widely recognized as the gold standard for lipid extraction due to its simplicity, speed, and high efficiency.^22^ The miscible chloroform/methanol/water system facilitates the interaction and complexation of the ions present, and subsequent dilution with water and chloroform enables isolation of the resulting HIP complex in the organic phase.^23^ Previous findings have shown that sulfonate groups exhibit superior precipitation efficiency compared to other tested anionic functional groups,^24^ which is consistent with the high precipitation efficiency observed for Cascade Blue hydrazide in this sulfonate-based system. As shown in Figure 1B, a charge ratio of 0.5:1 (DiA:Cascade Blue) resulted in 88% precipitation of the hydrophilic compound. Increasing the ratio to 1:1 led to a plateau in precipitation efficiency at 95%, so this ratio was selected for subsequent experiments. The partition coefficient log *P* between *n*-octanol/deionized water at pH ≈ 7 demonstrated that hydrophobic ion pairing between Cascade Blue and the lipophilic counterion DiA resulted in an 8130-fold increase in the Cascade Blue’s lipophilicity, as depicted in Figure 1C. Individual fluorescent components and their successful complexation were visualized under UV illumination (Figure 1D). Prior to complexation, Cascade Blue exhibited a blue color, while DiA appeared red. Upon complex formation and subsequent exposure to UV light, the resulting HIP complex displayed a distinct magenta color. Measurement of the fluorescence spectra revealed shifts in both the fluorescence excitation and emission spectra after the complexation (Figure 1E). This can be explained by changes in the dye’s local microenvironment due to interactions between the quaternary amonium of DiA and the sulfonate groups of Cascade Blue, resulting in a bathochromic shift.^25,26^ Molecular interactions were further investigated for a potential Förster resonance energy transfer (FRET) effect between the fluorophores. The FRET effect occurs as a result of energy transfer via dipole–dipole interactions, indicating molecular proximity within the HIP complex.^27^ Emission spectra of the individual compounds and the HIP complex were recorded using an excitation wavelength of 330 nm. Despite the pronounced spectral overlap between the donor and acceptor dyes, no evidence of FRET was observed in the emission spectra upon complexation (Figure 1F).

**Figure 1:**
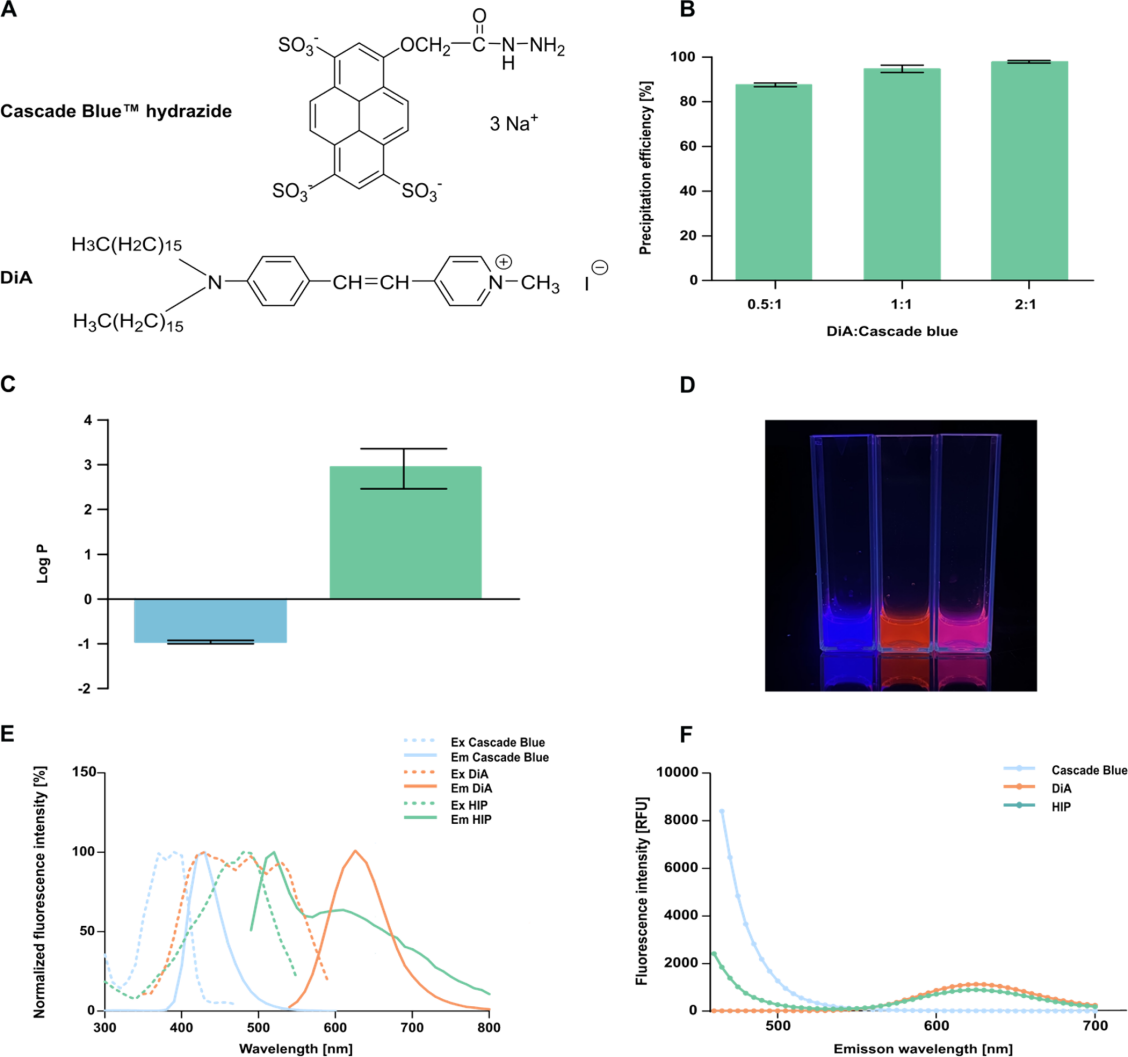
(A) Chemical structures of Cascade Blue hydrazide trisodium salt and DiA. (B) Precipitation efficiency [%] of Cascade Blue after hydrophobic ion pairing with the lipophilic counterion DiA at varying charge ratios. (C) log *P*_*n*‑octanol–water_ of (blue)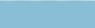 Cascade Blue and (green)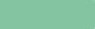 HIP complex at a 1:1 charge ratio. (D) Photos of Cascade Blue (blue), DiA (red), and the resulting HIP complex (magenta) in DMSO, under a UV lamp (Ex 330 nm). (E) Normalized fluorescence spectra of Cascade Blue (blue), DiA (orange), and HIP complex (green). Spectra were recorded in demineralized water containing 1% DMSO at room temperature. Each spectrum was normalized to its maximum intensity to facilitate comparison of spectral profiles. (F) Emission spectra of Cascade Blue (blue), DiA (orange), and HIP (green) in water with 1% DMSO at an excitation wavelength of 330 nm to evaluate potential FRET interactions. Data are presented as means ± standard deviation (*n* ≥ 3).

The dissociation of HIP is a crucial step in the drug release from carriers and can be triggered by the presence of competing ions in the surrounding medium. The rate of dissociation is influenced by several factors, including the ionic strength and pH of the medium and the concentration of counterions. Notably, an increased number of counterions leads to a slower dissociation.^13^ In simple salt solutions (NaCl, KCl, and CaCl_2_), only ∼25% of Cascade Blue dissociated after 24 h, whereas no release occurred in deionized water, as expected (Figure 2A). In contrast, experiments conducted in HBS buffer and various simulated physiological fluids demonstrated that the presence of multiple ions facilitates more effective dissociation than single-salt solutions and that the process is pH-dependent. Notably, the HIP was highly stable under acidic conditions (FaSSGF), while phosphate-containing intestinal fluids accelerated the dissociation, in line with previous reports.^24,28,29^ Overall, the dissociation followed the order FeSSIF > FaSSIF > HBS > FaSSGF (Figure 2B), highlighting the influence of the physiological environment on HIP stability.

**Figure 2:**
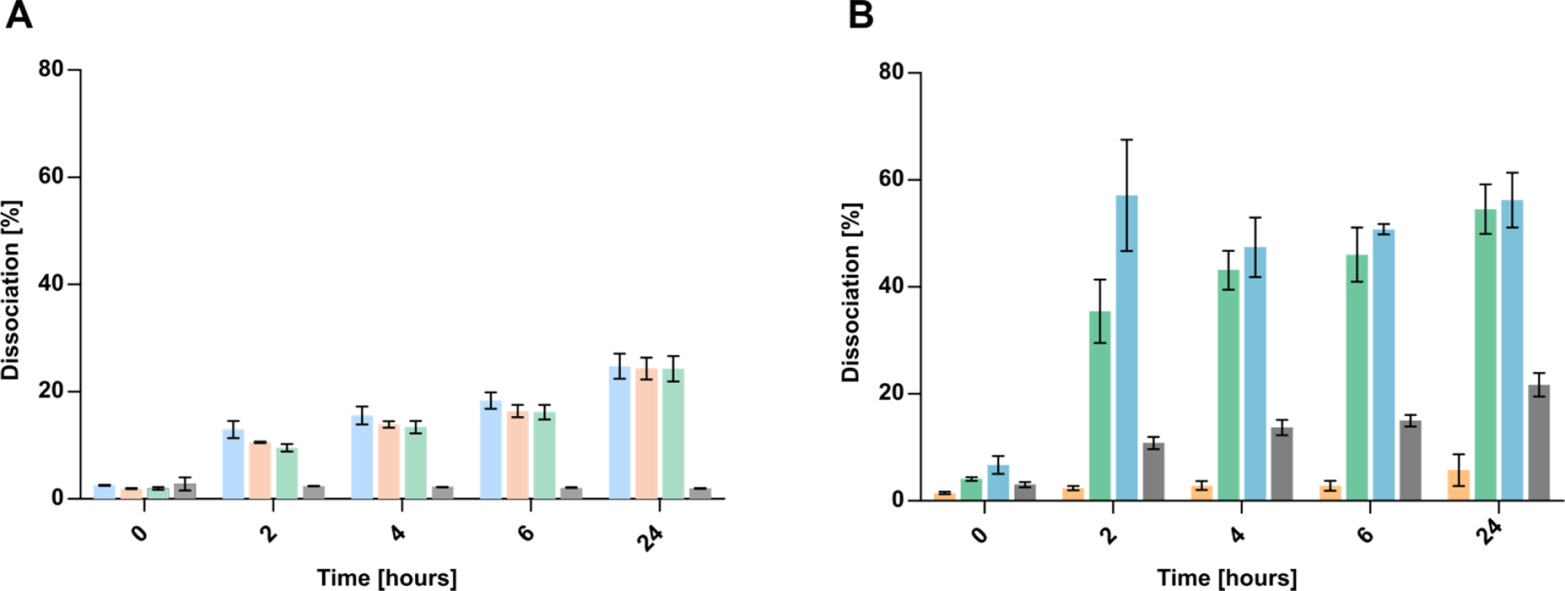
(A) Dissociation of HIP in (blue) 5% NaCl, (orange) 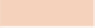
5% KCl, (green)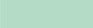 5% CaCl_2_, and (gray)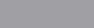 demineralized water. (B) Dissociation of HIP in (orange)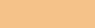 fasted-state gastric fluid (FaSSGF), (green)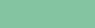 fasted-state intestinal fluid (FaSSIF), (blue)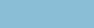 fed-state intestinal fluid (FeSSIF), and (gray)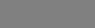 HBS buffer. Samples were incubated at 37 °C, 400 rpm, and light-protected for 24 h. The amount of Cascade Blue being released into the media was tested at predetermined time points. Data are presented as means ± standard deviation (*n* ≥ 3).

### 3.2 SEDDS, Nanoemulsion, and Liposome Characterization

Three different lipid-based formulations, summarized in Table 1, were prepared to study the cellular uptake mechanisms and to elucidate the fate of the fluorescent HIP complex after internalization. As shown in Table 2, the SEDDS formulation exhibited the smallest particle size, 64.41 ± 0.26 nm, after 1% dilution in HBS. In general, emulsions containing medium-chain triglycerides produce smaller droplet sizes than those containing long-chain fatty acids.^30^ Nanoemulsions, composed of long-chain oleic acid, DOPE, and Tween 80 (all containing long-chain fatty moieties), resulted in a particle size of 92.61 ± 1.27 nm. Liposomes prepared via the ethanol injection method formed large unilamellar vesicles with an average size of 175.03 ± 3.18 nm. All formulations exhibited a slight increase in particle size after the HIP complex was incorporated. The PDI ranged from 0.2 to 0.4, indicating a monodisperse distribution. The negative surface charge, measured in both SEDDS (−10.17 ± 0.35 mV) and nanoemulsion formulation (−32.40 ± 2.16), is most likely attributed to the presence of free fatty acids. These acids can migrate to the oil/water interface and, in their deprotonated state, influence the particle surface charge.^31,32^ Incorporation of the HIP complex increased the surface charge values for both SEDDS and nanoemulsion formulations. The liposomes were positively charged (+10.92 ± 0.76 mV) due to the presence of the cationic lipid DOTAP.

**Table 2:** Mean Particle Size (nm), Polydispersity Index (PDI), and Zeta Potential (mV) of Blank and HIP-Loaded SEDDS, Nanoemulsions, and Liposomes^a^

**formulation**	**size [nm]**	**PDI**	**zeta potential [mV]**
blank SEDDS	64.41 ± 0.26	0.185 ± 0.003	–10.2 ± 0.4
HIP + SEDDS	74.82 ± 0.27	0.236 ± 0.002	–0.6 ± 0.3
blank NE	92.61 ± 1.27	0.295 ± 0.025	–32.4 ± 2.2
HIP + NE	97.36 ± 0.51	0.363 ± 0.002	–9.5 ± 0.9
blank liposomes	175.03 ± 3.18	0.362 ± 0.006	10.9 ± 0.8
HIP + liposomes	176.37 ± 1.85	0.405 ± 0.012	9.4 ± 0.4

a All samples were diluted to 1% (v/v) using HBS buffer (or demineralized water for zeta potential measurements) and incubated at 37 °C prior to analysis.

### 3.3 Stability Studies

The stability of blank and HIP-loaded SEDDS, nanoemulsion, and liposomal formulations was evaluated in different media (HBS, FaSSGF, and FaSSIF) over a 24 h incubation period at 37 °C, as shown in Figure 3, and at 4 °C (Figure S1, Supporting Information). Both blank and HIP-loaded SEDDS formulations remained stable in HBS and FaSSGF, maintaining particle sizes <85 nm and PDI < 0.3. However, the blank SEDDS formulation became destabilized in the presence of FaSSIF after 24 h, with an increase in particle size to ∼261 nm and PDI > 1, indicating a broad size distribution and loss of colloidal stability. Bile salts present in FaSSIF are known to displace surfactants adsorbed at the surface of the oily droplets, thereby altering interfacial tension and thereby destabilizing the system.^33,34^ Conversely, the HIP-loaded SEDDS formulation exhibited a significant decrease in particle size to approximately 45 nm under the same conditions (Figure 3A,B). Both blank and HIP-loaded nanoemulsions exhibited good stability, with only minor fluctuations in size and PDI values across all media, as shown in Figure 3C,D. For liposomal formulations, both blank and HIP-loaded liposomes demonstrated stability in HBS. Incubation in FaSSGF decreased the particle size of blank liposomes, while HIP-loaded liposomes exhibited a slight increase in particle size when exposed to FaSSIF. Despite these changes, the PDI remained below or close to 0.4, indicating overall system stability (Figure 3E,F).

**Figure 3:**
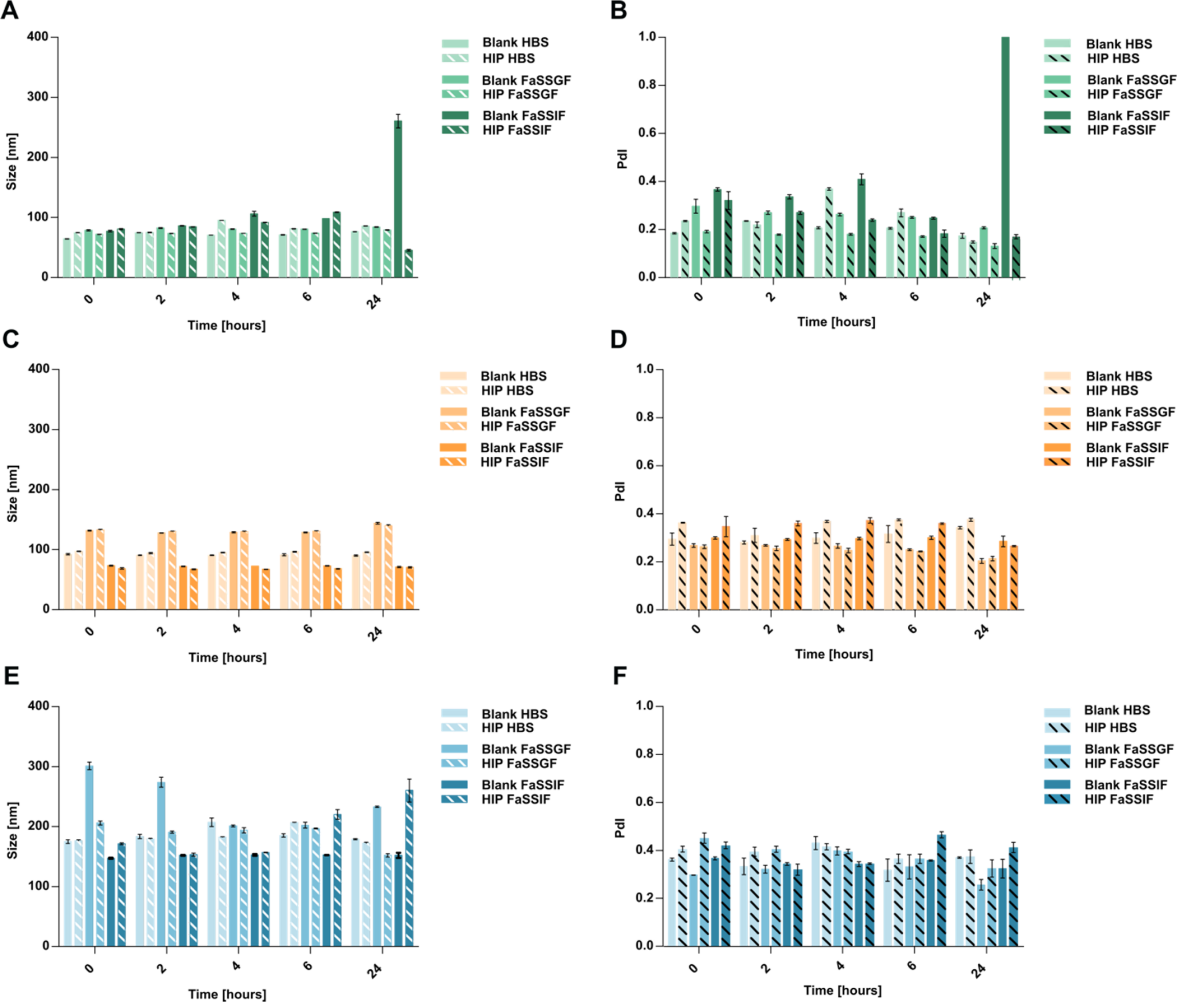
Stability studies of blank (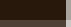
solid bar) and HIP-loaded (striped bar) SEDDS (green), nanoemulsions (orange), and liposomes (blue) in HBS, fasted-state gastric fluid (FaSSGF), and fasted-state intestinal fluid (FaSSIF). Prior to measurement, samples were diluted to 1% (v/v). Particle size (A, C, E) and polydispersity index (B, D, F) were analyzed immediately after dilution and following 2, 4, 6, and 24 h of incubation at 37 °C, 400 rpm. Data are presented as means ± standard deviation (*n* ≥ 3).

### 3.4 Drug Release Studies

A key factor governing the release of Cascade Blue hydrazide is its solubility in the release medium, which is substantially lowered by complexation into a HIP. As a result, the release involves three sequential steps: (i) diffusion of the intact HIP complex from the formulation, (ii) dissociation of Cascade Blue from the counterion, and (iii) diffusion of the free dye across the dialysis membrane.^35^ The stability and lipophilicity of the HIP complex significantly impact this process. Chamieh et al.^36^ demonstrated that an increased ionic strength in the release medium accelerates HIP dissociation, aligning with the dissociation behavior described in Section 3.1. Conversely, HIPs characterized by higher log *P* values are more strongly retained within oil-rich domains, resulting in sustained release.^37^ Consistent with this, the SEDDS formulation, owing to its higher lipid content and greater hydrophobic character, showed stronger retention of the HIP and consequently its slower release. Conversely, nanoemulsions and liposomes exhibited nearly identical release profiles over 48 h, indicating a lower capacity to sustain HIP retention (Figure 4A).

**Figure 4:**
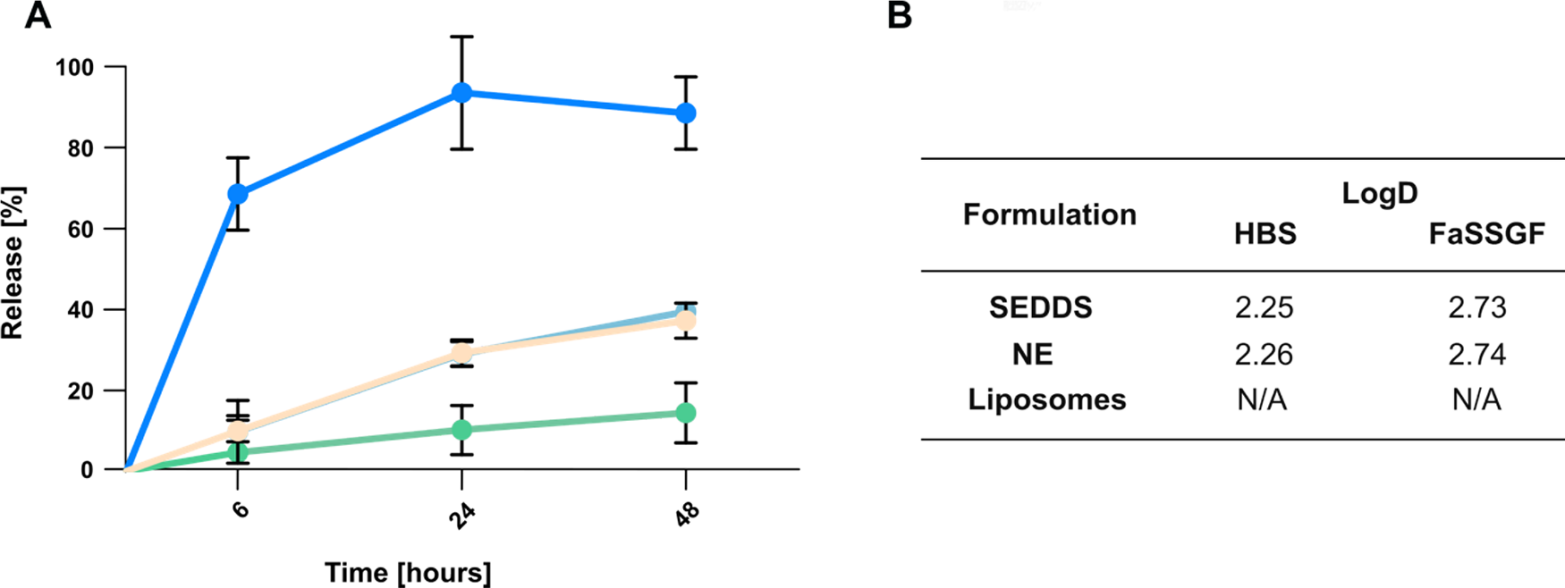
(A) Cumulative release of Cascade Blue hydrazide [%] from HIP-loaded SEDDS (green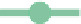), nanoemulsion (orange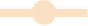), liposomes (light blue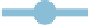), and free Cascade Blue hydrazide control (dark blue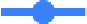). Samples were diluted with HBS buffer to a final concentration of 1% (v/v) and incubated at 37 °C, 100 rpm, for 48 h. Data are presented as means ± standard deviation (*n* ≥ 3). (B) log *D* between SEDDS/NE and HBS/FaSSGF. Results are expressed as means ± standard deviation (*n* ≥ 3).

The extent to which a drug is retained within a formulation is closely related to how it partitions between the lipid phase and the release medium. Therefore, log *D* values were determined for the SEDDS and nanoemulsion in HBS and FaSSGF (Figure 4B). An increase in log *D* corresponds to greater drug retention within the oily droplets, resulting in slower release from the formulation.^38^ However, in the case of the HIPs, dissociation in the presence of competing ions increases the concentration of the free, hydrophilic drug in the aqueous phase. This lowers the apparent log *D* and can lead to data misinterpretation.^39^ Consistent with this, the log *D* value of the HIP measured in FaSSGF was higher than in HBS, correlating well with the dissociation behavior discussed in the Section 3.1.

### 3.5 Cytotoxicity Studies

The cytotoxicity of both the blank and the HIP-loaded formulations was evaluated in the Caco-2 cell line following 4 and 24 h incubation periods across concentrations ranging from 0.25% to 0.005% (Figure 5). The strongest reduction in cell viability was observed for nanoemulsions, likely due to the synergistic effects of surfactants and cosolvents leading to membrane disruption.^40,41^ Liposomal carriers displayed moderate cytotoxicity at intermediate concentrations, which can be attributed to the electrostatic interaction between their positively charged surfaces and negatively charged cell membranes.^42^ In contrast, SEDDS containing Solutol HS-15,^43^ a surfactant with reported low toxicity, showed the highest cell viability across all tested concentrations. Notably, HIP loading did not markedly affect cytotoxicity compared to blank carriers. This indicates that the carrier type, rather than the HIP complex, determines cell compatibility. After 24 h of incubation, all formulations maintained ≥90% cell viability at or below 0.01%, suggesting their suitability for further studies. Additionally, the cytotoxicity of selected inhibitors was evaluated (Figure S2A, Supporting Information).

**Figure 5:**
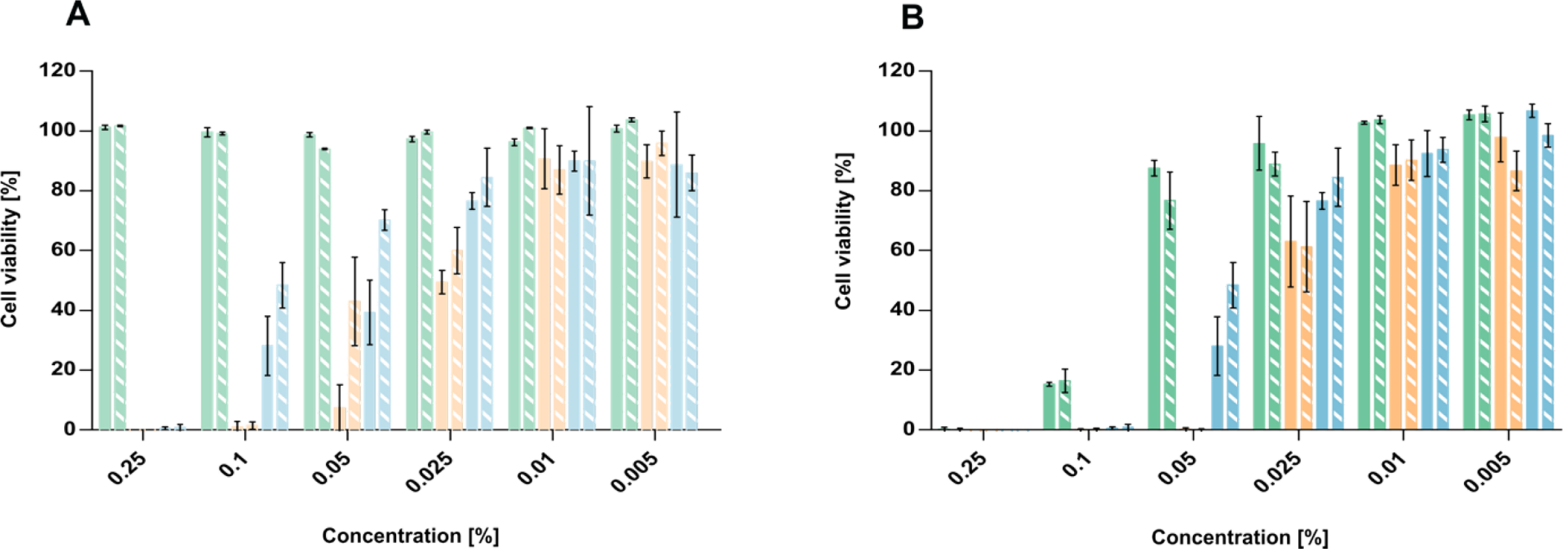
Cell viability [%] of Caco-2 cells (A) after 4 h of incubation with blank ((solid green)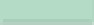 SEDDS, (solid orange)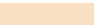 nanoemulsions, and (solid blue)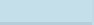 liposomes) and HIP-loaded ((striped green)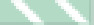 SEDDS, (striped orange)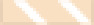 nanoemulsions, and (striped blue) liposomes) formulations at different concentrations at 37 °C and (B) after 24 h of incubation with blank ((solid green) SEDDS, (solid orange) nanoemulsions, and (solid blue)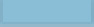 liposomes) and HIP-loaded ((striped green)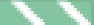 SEDDS, (striped orange)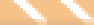 nanoemulsions, and (striped blue)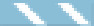 liposomes) formulations at indicated concentrations at 37 °C. Data are presented as means ± standard deviation (*n* ≥ 3).

### 3.6 From Uptake to Fate: Intracellular Behavior of HIP-Loaded Nanocarries

#### 3.6.1 Cellular Uptake

The internalization of the formulations was evaluated at 37 and 4 °C in the absence or presence of selected chemical inhibitors (Figure 6). Chlorpromazine (30 μM) was used to inhibit clathrin-mediated endocytosis by disrupting clathrin assembly at the plasma membrane.^44^ Methyl-β-cyclodextrin (5 mM) sequesters membrane cholesterol, compromising cholesterol-rich lipid-raft integrity and preventing caveolae formation,^45,46^ thereby inhibiting claveolae-mediated uptake. Incubation at 4 °C was used to block energy-dependent processes; however, membrane fusion can still occur under these conditions.^7^ To discriminate surface-bound particles from internalized formulations, trypan blue was applied as an extracellular quenching agent.^47^ At 37 °C, chlorpromazine reduced cellular uptake by 9.5%, 12.75%, and 8.95% for SEDDS, nanoemulsions, and liposomes, respectively. In contrast, methyl-β-cyclodextrin increased the uptake by 7.92%, 4.35%, and 11.15% for the same formulations. Similar effects have also been reported in a study, where nystatin pretreatment (another caveolae-inhibiting agent) enhanced nanoparticle uptake in Caco-2 cells.^48^ Incubation at 4 °C markedly suppressed the internalization of all formulations across conditions, indicating that the uptake mechanism is primarily energy-dependent. No evidence of fusion-driven uptake was observed for liposomes at the tested concentration; however, at a higher liposome concentration (0.025% w/v), pronounced internalization persisted at 4 °C, suggesting that membrane fusion may complement endocytosis at elevated concentrations (Figure S3, Supporting Information). Huth et al.^49^ showed in their study that fusion of liposomes was complementary to endocytic internalization. Based on their results, different mechanisms are involved in the internalization of liposomes in parallel, and several factors affect the mechanism of internalization, including liposomal composition, particle size, surface charge, and targeted cells.

**Figure 6:**
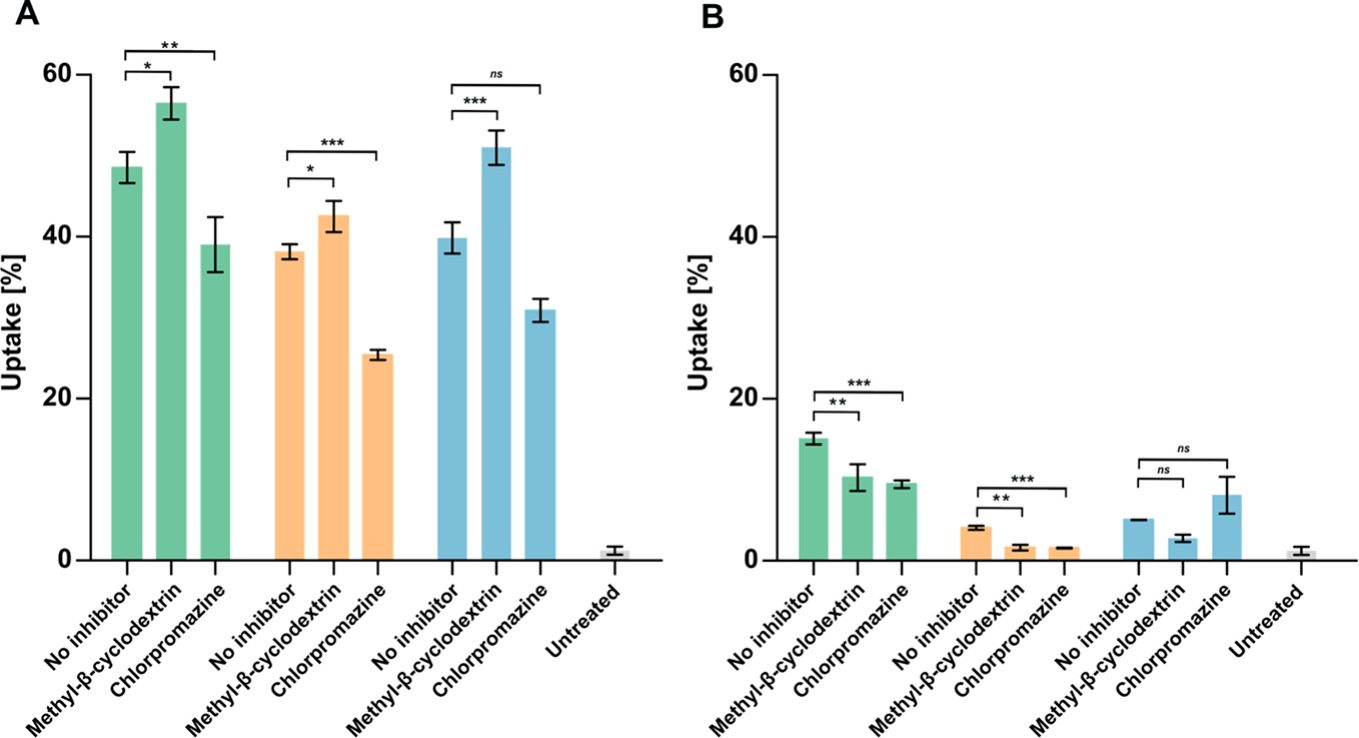
Cellular internalization [%] of HIP-loaded (green) SEDDS, (orange)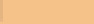 nanoemulsions, and (blue)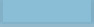 liposomes by Caco-2 cells after 2 h of incubation at (A) 37 °C and (B) 4 °C. Where indicated, the cells were pretreated with methyl-β-cyclodextrin (5 mM) and chlorpromazine (30 μM) for 45 min prior to sample application. Untreated cells (washed with HBS and not exposed to any formulation) served as the baseline control. Data are presented as means ± standard deviation (*n* ≥ 3). Significant differences compared to formulations without an inhibitor are indicated as follows: *p < 0.05; **p < 0.01; ***p < 0.001.

#### 3.6.2 Endosomal Escape Studies

In this study, the hemolysis assay was employed as a surrogate model to evaluate the interaction of the investigated formulations with endosomal membranes (Figure 7).^50^ This approach enabled us to assess their potential to induce membrane destabilization, which may subsequently promote the release of the encapsulated fluorescent dyes. After 4 h of incubation (Figure 7A), both the nanoemulsion and the liposomal formulations induced substantial hemolysis, with comparable levels observed between the two systems at the highest tested concentrations. Oleic acid, present in both formulations, is known for its ability to insert into lipid membrane bilayers, causing hemolysis even at low concentrations.^51^ In contrast, the SEDDS formulation exhibited negligible hemolytic activity at all concentrations, indicating minimal interaction with the erythrocyte membrane. After 24 h of incubation (Figure 7B), a clear, time-dependent increase in hemolysis was observed for the nanoemulsion, which remained the most disruptive formulation. Liposomes also induced pronounced hemolysis, though to a slightly lower extent than the nanoemulsion. This enhanced hemolytic effect is attributed not only to the presence of oleic acid but also to their slightly positive surface charge, which facilitates electrostatic interactions with the negatively charged erythrocyte membrane, promoting local destabilization and disruption of the membrane.^52,53^ In contrast, the SEDDS formulation showed negligible hemolytic activity even after prolonged exposure, indicating a favorable safety profile with minimal membrane disruptive potential. Additionally, the hemolytic effect of selected inihibitors on membranes was investigated (Figure S2B, Supporting Information).

**Figure 7:**
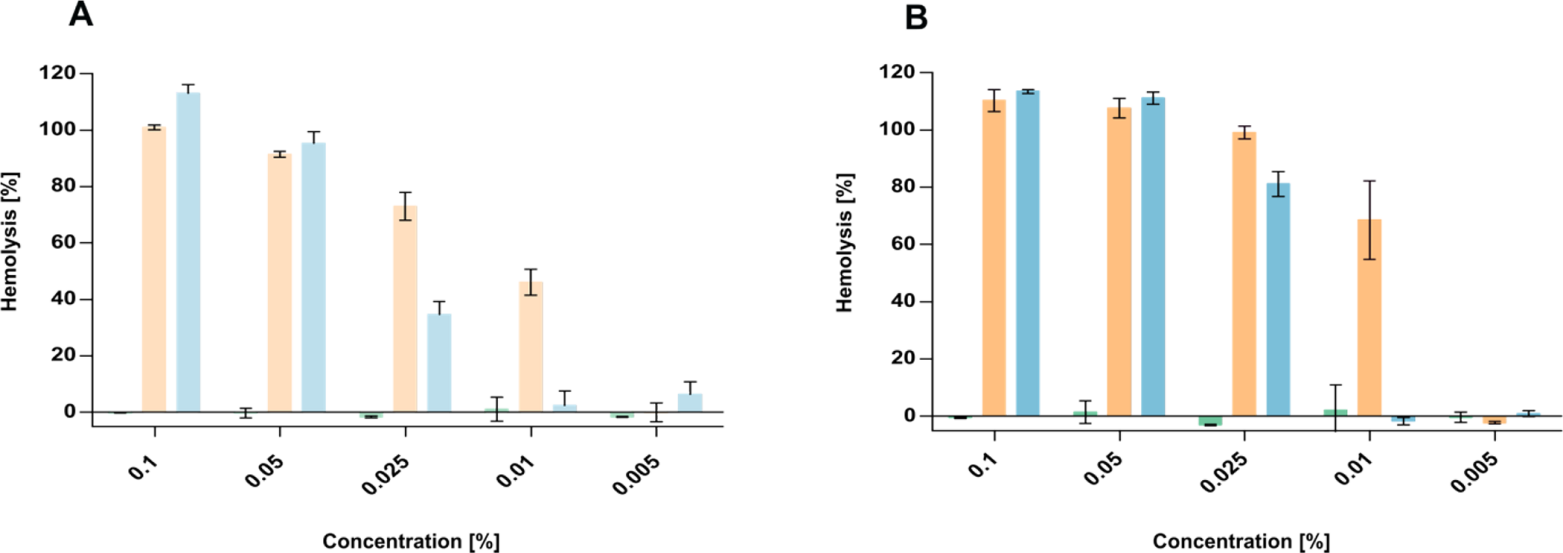
Hemolysis [%] of erythrocytes after incubation at 37 °C with blank formulations at varying concentrations: (A) after 4 h ((green)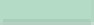 SEDDS, (orange)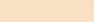 nanoemulsions, 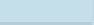 and (blue) liposomes) and (B) after 24 h ((green)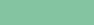 SEDDS, (orange)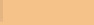 nanoemulsions, and (blue)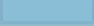 liposomes). Data are presented as means ± standard deviation (*n* ≥ 3).

#### 3.6.3 Intracellular Trafficking of the HIP Complex

CLSM was employed to investigate the intracellular fate of the fluorescent probes in Caco-2 cells at 37 and 4 °C, in the presence and absence of selected inhibitors (Figure 8). DiA (appeared as red), a lipophilic dialkylaminostyryl membrane dye, is able to easily bind with cell membranes,^10^ whereas Cascade Blue (appeared as blue) is a hydrophilic dye, which alone is cell-impermeant (supplier’s information). The appearance of magenta regions indicates channel overlap, marking sites where both probes remained associated within the HIP complex.

**Figure 8:**
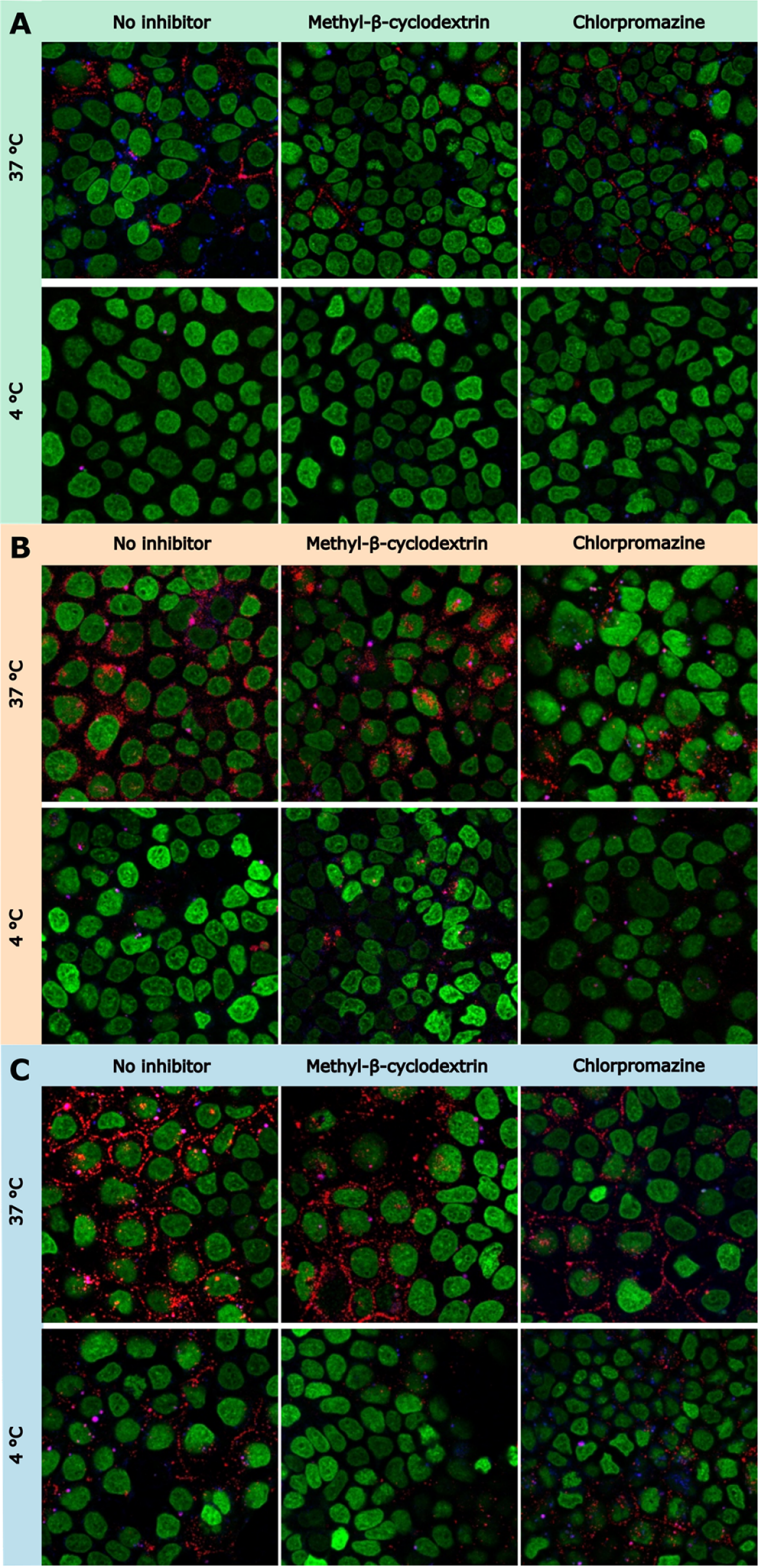
Overlay confocal micrographs of Caco-2 cells after 2 h of incubation with (A) SEDDS, (B) nanoemulsion, and (C) liposomal HIP-loaded formulations at 37 or 4 °C, with or without endocytic pathway modulators. Columns show no inhibitor (control), chlorpromazine (30 μM; clathrin-mediated endocytosis inhibitor), and methyl-β-cyclodextrin (5 mM; cholesterol sequestration/lipid-raft–caveolae disruption). For inhibitor conditions, cells were preincubated for 45 min before formulation addition. DiA is visualized in red; Cascade Blue in blue; magenta puncta indicate intact HIP complexes internalized within vesicular structures; NucSpot-650-labeled nuclei appear green.

Upon internalization of SEDDS formulation (Figure 8A), dissociation of the HIP complex was apparent. DiA predominantly diffused into the plasma membrane, and the punctuate signal of Cascade Blue indicated its confinement in intracellular vesicles. These observations align with the slowest drug release measured for the SEDDS, which was previously discussed in Section 3.4 and with low endosomal escape observed for this formulation. Methyl-β-cyclodextrin resulted in a more visible reduction in cellular uptake compared to chlorpromazine. Incubation at 4 °C completely inhibited uptake under all tested conditions, confirming that the transport process was energy-dependent. In the nanoemulsion condition (Figure 8B), DiA was delivered to nuclei’s close proximity and accumulated in the perinuclear membranes, while the bright punctate signal of Cascade Blue present in endosomes was no longer detectable. The absence of a detectable Cascade Blue signal likely indicates that, following endosomal escape, the hydrophilic dye rapidly diffused throughout the cytoplasm, resulting in substantial dilution and a fluorescence intensity below the detection limit of the microscope. Similar observations have been reported previously, where diffuse cytosolic distribution led to undetectable fluorescence despite successful endosomal escape.^54^ This interpretation is consistent with the high endosomal escape efficiency obtained for these formulations. Residual magenta regions indicate vesicles that still contained intact HIP. Cellular uptake of the nanoemulsion was more sensitive to chlorpromazine than to methyl-β-cyclodextrin and was likewise suppressed at 4 °C. Following exposure to liposomes, the HIP was predominantly dissociated (Figure 8C). DiA redistribution to the plasma membrane was more pronounced than in the case of SEDDS formulation, whereas Cascade Blue no longer formed blue intracellular puncta under most conditions. At 4 °C, uptake was markedly reduced. However, DiA colocalization in the plasma membrane was still partially visible, and magenta puncta were observed in HBS- and chlorpromazine-treated cells. Detectable blue puncta showed that Cascade Blue remained encapsulated within endocytic vesicles. These observations suggest the presence of limited energy-independent processes such as lipid mixing or membrane fusion. In both cases, nanoemulsions and liposomes, Cascade Blue was able to escape endosomes into the cytosol.

## 4 Conclusions

This study aimed to compare the influence of lipid-based nanocarriers on the cellular uptake and intracellular fate of a dual-fluorescent hydrophobic ion pair. By pairing the hydrophilic dye Cascade Blue with the lipophilic probe DiA, we established a mechanistic model for hydrophilic drug surrogates that enabled the simultaneous visualization of both components and provided insights into their intracellular behavior after delivery.

Our results showed that carrier composition had a pronounced effect on both uptake efficiency and intracellular localization. While SEDDS were stable and biocompatible, the hydrophilic dye remained mainly confined to vesicular compartments, suggesting limited endosomal escape. In contrast, nanoemulsions and liposomes showed high endosomal escape as indicated by the quantitative assay, although no detectable Cascade Blue fluorescence was observed by confocal microscopy. This absence of visible signal likely reflects the rapid diffusion and dilution of the released dye within the cytoplasm following endosomal escape, resulting in fluorescence below the detection limit. For liposomes, which contained fusion-promoting lipids, elevated concentrations (0.025%) led to noticeably higher uptake even at 4 °C, suggesting that complementary fusion-mediated internalization might also occur alongside endocytosis.

Overall, these findings demonstrate that even subtle differences in formulation composition can alter cellular uptake pathways and intracellular distribution. The dual-fluorescent HIP system proved to be a sensitive and versatile mechanistic tool for visualizing the fate of hydrophilic drug surrogates and may support the design of lipid-based carriers optimized for cytosolic delivery of hydrophilic compounds.

## Supplementary Material



## Notes

Stability studies of blank and HIP-loaded SEDDS, nanoemulsions, and liposomes (blue) in HBS, fasted-state gastric fluid (FaSSGF), and fasted-state intestinal fluid (FaSSIF) incubated at 4° (Figure S1); cell viability of Caco-2 cells and hemolysis of erythrocytes [%] after incubation with selected inhibitors (Figure S2); cellular internalization of liposomes (0.025%) by Caco-2 cells after 2 h of incubation at 37 and 4 °C (Figure S3) (PDF)
