# Supplementary material for: Intracellular Fate of a Dual-Fluorescent Hydrophobic Ion Pair: Comparison of Lipid-Based Nanocarriers
Source: Mol Pharm. 2026 Feb 12;23(3):1929–41. doi: 10.1021/acs.molpharmaceut.5c01633 (PMC12958344; doi:10.1021/acs.molpharmaceut.5c01633)
Supplement: Supplementary file 1 [file mp5c01633_si_001.pdf]

Supporting information

Intracellular Fate of a Dual-Fluorescent Hydrophobic Ion Pair: Comparison of Lipid-Based Nanocarriers

Gabriela Koutná[1,2], Lena Werner[1], Martyna Truszkowska[1], Luca Maurice Richter[1], Kateřina Kubová[2], Andreas Bernkop-Schnürch[1*]

[1] Department of Pharmaceutical Technology, Institute of Pharmacy, University of Innsbruck, Innrain 80/82, Innsbruck 6020, Austria; Lena.Werner@uibk.ac.at (L.S.W.); matynatruszkowska@gmail.com (M.T.); Luca.Richter@uibk.ac.at (L.M.R.)

[2] Department of Pharmaceutical Technology, Faculty of Pharmacy, Masaryk University, Palackého třída 1946/1, Brno 612 00, Czech Republic; 507108@mail.muni.cz (G.K.); kubovak@pharm.muni.cz (K.K.)

*** Correspondence:** Andreas.Bernkop@uibk.ac.at (A.B.-S.)

1.1 Stability studies at 4 °C

The stability of HIP-loaded and blank formulations was evaluated at 4 °C for 24-hour period. No significant changes in particle size or polydispersity index were observed across the tested systems, confirming colloidal stability under storage conditions. SEDDS maintained a consistently small droplet size (~97 nm) with a PdI < 0.4. Nanoemulsions remained stable in both particle size and PdI, with sizes ranging between ~90–120 nm depending on the incubation medium. Liposomes were stable in HBS buffer and FaSSGF, while incubation in FaSSIF led to a slight increase in particle size over 24 h, consistent with observations at 37 °C. HIP loading did not compromise stability, as HIP-loaded formulations showed comparable size and surface charge to their blank counterparts. These results confirm that both blank and HIP-loaded lipid-based carriers remain physically stable when stored at 4 °C, supporting their suitability for subsequent in vitro studies.

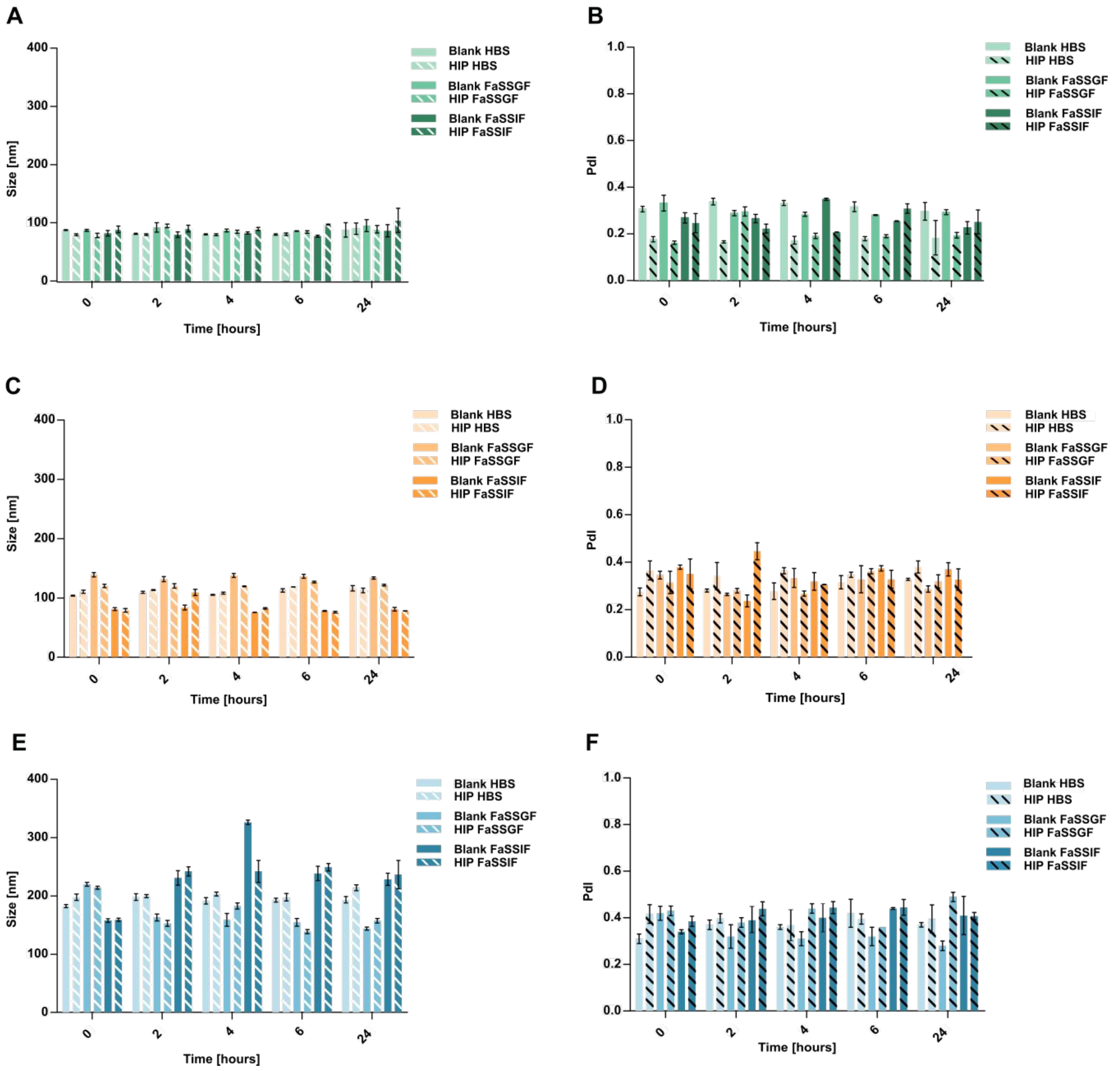

Fig. S1: Stability studies of blank (solid bar) and HIP-loaded (striped bar) SEDDS (green), nanoemulsions (orange) and liposomes (blue) in HBS, fasted state gastric fluid (FaSSGF) and fasted state intestinal fluid (FaSSIF). Prior to measurement, samples were diluted to 1% (v/v). Particle size (A, C, E) and polydispersity index (B, D, F) were analysed immediately after dilution and following 2, 4, 6 and 24 hours of incubation at 4 °C, 400 rpm. Data are presented as means ± standard deviation ($n \geq 3$).

1.2 Cell viability and hemolysis

To ensure that the observed effects on cellular uptake were not confounded by inhibitor-induced toxicity, the cytotoxicity and hemolytic potential of chlorpromazine and methyl-β-cyclodextrin were evaluated systematically. Chlorpromazine exhibited high cell viability (>80%) across all tested concentrations and caused no measurable hemolysis. In contrast, methyl-β-cyclodextrin exhibited concentration-dependent reductions in viability and induced significant hemolysis only at concentrations above 10 mM. These results confirm that the concentrations used in the uptake experiments–30 µM of chlorpromazine and 5 mM of methyl-β-cyclodextrin–did not compromise cell viability or erythrocyte integrity[1,2].

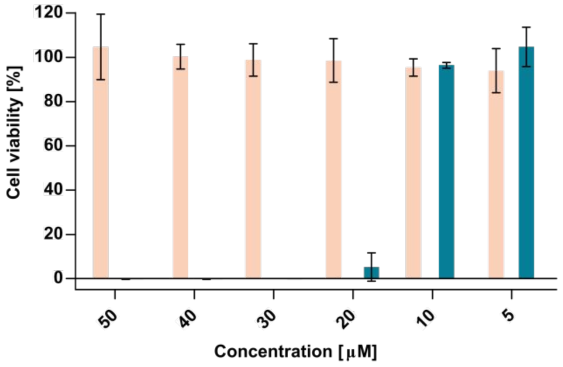

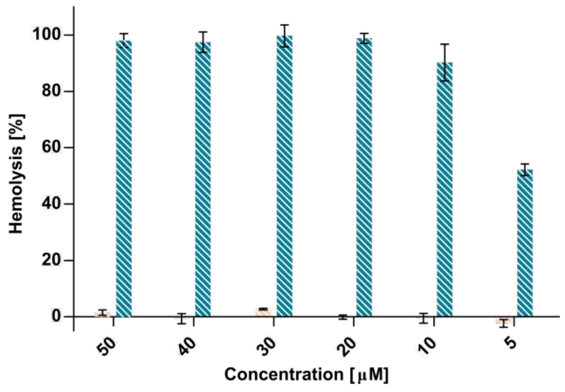

Fig. S2: A: Cell viability of Caco-2 cells after incubation with selected inhibitors: chlorpromazine (µM); methyl-ß-cyclodextrin (mM) in different concentrations for 2 h at 37 °C. B: Haemolysis [%] of erythrocytes after incubation with selected inhibitors: chlorpromazine (µM); methyl-ß-cyclodextrin (mM) in different concentrations for 2h at 37°C. Data are presented as means ± standard deviation (n ≥ 3).

1.3 Cellular uptake

Cellular uptake studies confirmed that liposomes are predominantly internalized via energy-dependent mechanisms, as the uptake was markedly reduced at 4 °C. However, at higher concentration of 0.025% (w/v), liposomes still exhibited a measurable degree of uptake under these energy-blocked conditions. This residual signal strongly suggests a fusion-driven contribution to internalization, consistent with the fusogenic nature of the lipid composition. Such fusion-mediated entry would enable direct delivery of membrane-associated DiA to the plasma membrane while simultaneously promoting cytosolic access of the paired Cascade Blue™. These findings support the dual role of liposomes: while endocytosis remains the primary pathway of uptake, fusion events complement this process and may enhance intracellular delivery efficiency [3]. This mechanism is particularly relevant for the design of lipid-based carriers where bypassing endosomal entrapment is desired.

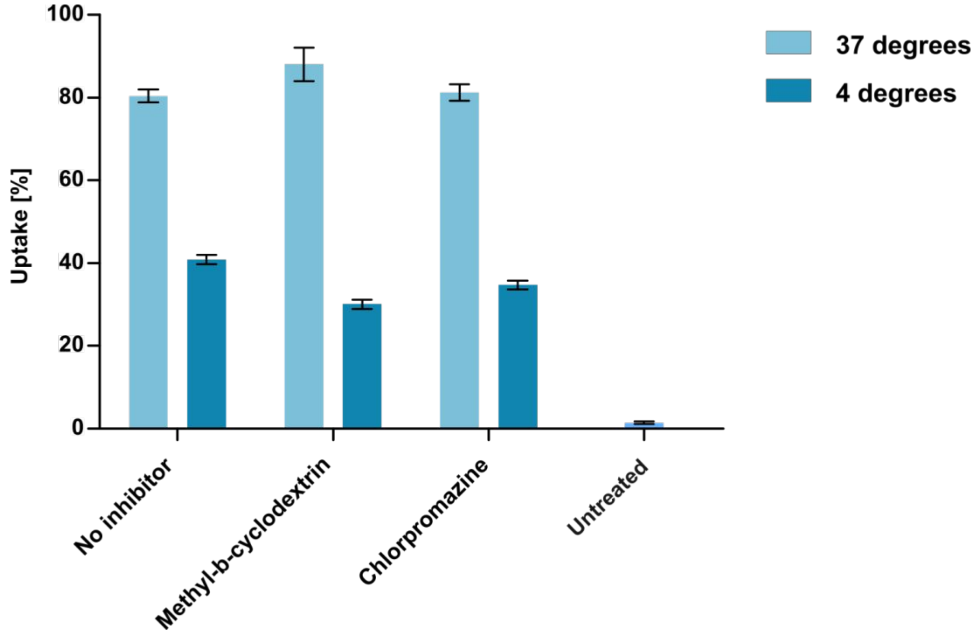

Fig. S3: Cellular internalization of liposomes 0.025% by Caco-2 cells after 2 hours of incubation at 37 °C and 4°C. Where indicated, the cells were pre-treated with methyl-ß-cyclodextrin (5 mM) and chlorpromazine (30 µM) for 45 minutes prior to sample application. Untreated cells (washed with HBS and not exposed to any formulation) served as the baseline control. Data are presented as means ± standard deviation (n ≥ 3).

References

(1) Danthi, P.; Chow, M. Cholesterol Removal by Methyl-β-Cyclodextrin Inhibits Poliovirus Entry. *J Virol* **2004**, *78* (1), 33–41. https://doi.org/10.1128/JVI.78.1.33-41.2004.

(2) Ng, C. T.; Tang, F. M. A.; Li, J. J.; Ong, C.; Yung, L. L. Y.; Bay, B. H. Clathrin-Mediated Endocytosis of Gold Nanoparticles *In Vitro*. *The Anatomical Record* **2015**, *298* (2), 418–427. https://doi.org/10.1002/ar.23051.

(3) Huth, U. S.; Schubert, R.; Peschka-Süss, R. Investigating the Uptake and Intracellular Fate of pH-Sensitive Liposomes by Flow Cytometry and Spectral Bio-Imaging. *Journal of Controlled Release* **2006**, *110* (3), 490–504. https://doi.org/10.1016/j.jconrel.2005.10.018.
